# Occupation as an associated risk factor for leptospirosis

**DOI:** 10.3389/fpubh.2026.1810977

**Published:** 2026-08-17

**Authors:** Cassiana Dahlke Machado, Louise Bach Kmetiuk, Alessandra Cristiane Sibim, Leandro Meneguelli Biondo, Wagner Antonio Chiba de Castro, Alexander Welker Biondo

**Affiliations:** 1 Zoonosis Control Center, City Secretary of Health, São José dos Pinhais, Brazil; 2 Center of Epidemiology, City Secretary of Health, Curitiba, Brazil; 3 Latin-American Institute of Life and Nature Sciences, Federal University for Latin American Integration (UNILA), Foz do Iguaçu, Brazil; 4 Interdisciplinary Graduate Studies, University of British Columbia, Kelowna, BC, Canada; 5 Department of Veterinary Medicine, Federal University of Paraná State (UFPR), Curitiba, Brazil

**Keywords:** datasus, environmental risk factors, *Leptospira*, occupational risk factors, retrospective analysis

## Abstract

**Introduction:**

Although several studies have described occupation as an associated risk factor for leptospirosis, most have reported absolute case counts without normalization by the general population, service attendance, or notification capacity.

**Methods:**

Accordingly, this study assessed the Brazilian National Surveillance System (SINAN) from 2007 to 2022, examining gathered leptospirosis cases analyzed by occupation as an associated risk factor within the working-age population. To ensure homogeneous attendance and notification capacity, we focused our analysis on the 100 largest Brazilian cities, utilizing censitary working occupation data per region as the denominator for normalization. Non-active populations (students and retirees) were excluded from the denominators to prevent bias dilution. Occupational groups were categorized into low, medium, and high risk for leptospirosis.

**Results:**

Overall, the occupations with the highest absolute frequency included farmers (12.0%), construction workers (5.8%), and recycling material collectors (4.6%), with the work environment (75.5%) identified as the most common site of infection. The highest normalized occurrence of leptospirosis was reported in southern Brazil, and high-risk occupational groups showed significantly higher infection rates compared to lower-risk categories (*p* < 0.001). Furthermore, professional groups with higher exposure loads presented greater frequencies of severe clinical outcomes, including renal failure, jaundice, and higher case fatality rates. The present study suggests occupation as an important predictor of leptospirosis, varying across regions according to local profe sional frequencies. In this scenario, the Brazilian national surveillance system serves as an effective indicator for public health actions and policies, enabling the dynamic monitoring of occupational exposure over time. Finally, occupation must systematically be analyzed as a predictor after normalizing for professional frequency, ensuring equitable access to healthcare assistance and standardized institutional notification.

## 1 Introduction

Human leptospirosis, which is caused by pathogenic *Leptospira* spp. and characterized by high treatment, morbidity, and lethality costs, is a major challenge for global public health (1). Leptospirosis, a widespread zoonotic infection in tropical and subtropical regions, is one of the most neglected tropical diseases worldwide (1). Human leptospirosis has been estimated at around 1.03 million annual cases and 58,900 deaths (2, 3), with an infection rate of 5 cases in endemic regions and 14 cases in outbreaks per 100,000 habitants (4). As the overall 9% mortality may reach 50% in severe leptospirosis cases, the global death burden highlights its public health (5). Considered as the most infected country in the Americas, Brazil has accounted for 40.2% of reported cases on the continent (2), with a yearly average of 3,846 cases between 2007 and 2017 (6). Similar to several other endemic countries, human leptospirosis notification is mandatory in Brazil, with a national surveillance system coordinated by the Ministry of Health (7).

Although accidental hosts have low efficiency in perpetuating the infection, human leptospirosis may be considered endemic and become an epidemic owing to massive exposure to common infection sources, particularly in workplaces (4). Leptospirosis transmission mainly occurs indirectly through contact with contaminated water and soil, caused by environmental spreading (8). Workers in direct contact with contaminated water and soil, such as workers in water treatment plants, sewer systems, construction, fisheries, and agriculture, are more likely to be exposed to occupational risks (9).

Accordingly, human leptospirosis has been considered an important occupational health concern worldwide, affecting workers in different fields such as janitors, sewer cleaners, sewage maintainers, street sweepers, trash collectors, peasants, agriculture and livestock farmers, rice planters, veterinarians, slaughterhouse employees, butchers, animal keepers, fishermen, miners, laboratory and military personal, and firefighters (10). Moreover, people living or working in flooded urban areas, under precarious sanitary infrastructure, or in contact with water, mud, or sewers are reportedly more exposed to leptospirosis (2, 11).

Leptospirosis remains a significant occupational public health concern, particularly in Brazil and other tropical countries, mostly because of the daily exposure to contaminated water, mud, sewers, and recycled materials. Although several studies have described occupation as an predictor for leptospirosis, most have only reported absolute cases, without normalization by the general population, attendance, or notification (12).

The classification of leptospirosis as a neglected disease has been intrinsically linked to the scarcity of epidemiological data which considers the overall workforce as a denominator for occupational risk. The absence of normalized incidence rates may have biased the real disease risk (frequency of exposure patterns) faced by specific categories; for example, low absolute case number but relatively high occupational exposure (11, 13). This lack of information, exacerbated by surveillance services focused only on severe cases, may have historically impaired effective occupational health public policies. Thus, breaking such perpetuated invisibility cycles may facilitate the proper allocation of resources and implementation of preventive measures in the most affected productive sectors (14, 15).

A recent study in Brazil identified occupational groups vulnerable to leptospirosis by applying Odds Ratio models among suspected cases, highlighting the difficulty in establishing actual incidence rates due to the lack of data on population frequencies of each professional group leading to biased outcomes (16). In addition, a survey in Malaysia focusing on specific groups (e.g., urban workers), indicated a lack of normalized risk analyses for the overall workforce of large centers (10). Thus, the methodology proposed here aims to fill this normalization gap by applying a frequency denominator of occupation estimates based on the updated Brazilian census data (17).

To mitigate reporting gaps and establish a robust normalization model, this study analyzed the 100 largest Brazilian cities. Although leptospirosis has traditionally been associated with rural and agricultural contexts, focusing on these large urban centers has provided a strategic advantage in terms of data reliability and surveillance infrastructure, effectively capturing both urban exposure and regional rural cases referred to these centers. The 100 municipalities are distributed across all 26 Brazilian states and the federal capital, ensuring wide geographic representation. Furthermore, these cities function as regional health centers, offering essential diagnostic support and specialized treatment to smaller adjacent municipalities (3, 18). Consequently, the analysis of these centers has allowed the model to utilize higher-capacity surveillance data while reflecting the epidemiological dynamics and occupational risks of their respective macroregions (19, 20).

In this context, the Compulsory Notifiable Diseases Information System (SINAN) represents the most strategic tool to address notification gaps in Brazil. Beyond serving as the official national surveillance system, SINAN provides the essential framework for systematic reporting, transparency, and monitoring of occupational associated risk factors over time. Emphasizing SINAN as a central instrument strengthens the rationale for this study, which seeks to evaluate occupational exposure to leptospirosis using standardized national data and to highlight the importance of improving notification practices for worker health. Thus, this study assessed the SINAN database from 2007 to 2022, with leptospirosis cases gathered, presented, and analyzed by occupation, to identify potential statistical associations with professional activities across the 100 largest cities using censitary working occupations per Brazilian region as normalization data.

## 2 Materials and methods

### 2.1 Dataset source

This was a retrospective assessment of notified leptospirosis cases registered by the SINAN, officially requested from 2007 to the most available updated full year (2022) and approved and undisclosed (to ensure human ethics compliance) by the Brazilian Ministry of Health in January 2024 (protocol number 23546.110497/2023–14). Included as a systemic disease with compulsory notification since 1993, all isolated or outbreak leptospirosis cases should be immediately reported online (within 24 h) to the city, state, and federal health authorities, along with an epidemiological questionnaire, as currently established and regulated by Federal Law No. 204/2016 (21). Epidemiological data included undisclosed patient information, clinical history and symptoms, occupation, laboratory results, likely infection location, final leptospirosis diagnosis (confirmed or ruled out on a laboratory or clinical-epidemiological basis), and disease outcome (cure or death).

According to the Brazilian Ministry of Health, a suspected leptospirosis case is defined as an individual presenting with fever, headache, and myalgia accompanied by at least one of the following criteria: 1. suggestive epidemiological history in the 30 days before clinical onset, including physical contact with flooding areas, mud, or sewer, exposure to flooding, sewer, or trash, epidemiological association (confirmed by laboratory results) with a confirmed case, household, or work in leptospirosis risk areas; or 2. Presence of at least one of the following signs: conjunctival suffusion, icterus, elevated bilirubin, signs of acute renal failure, or hemorrhagic symptoms (22).

Suspected cases were confirmed using clinical laboratories or epidemiological criteria. Clinical laboratory testing requires a suspicious case associated with one or more results, including ELISA-IgM positive test with seroconversion by Microagglutination Test (MAT), between two samplings with 14-to 60-day intervals; or at least a 4-fold increase in antibody titer by MAT (two samplings), or a sample with at least 800 titer, isolation of Leptospira in blood, and detection of Leptospira DNA by PCR in blood samples with anticoagulants (except heparin) in patients within 10 days of symptom onset. In the case of death, immunohistochemistry was positive for leptospirosis or other anatomic-pathological analyses with positive silver staining. Confirmed clinical and epidemiological cases were defined as suspicious cases with fever and hepatic, renal, or vascular alterations combined with epidemiological history, but without laboratory confirmation for any reason, likely not sampled for specific testing, or a negative result of serological testing sampled before the seventh day of the disease (22).

### 2.2 Dataset classification for normalization

The Brazilian Institute of Geography and Statistics (BIGS) is recognized as the main official data provider in Brazil. One fundamental duty of the BIGS is the Continuous National Household Sample Survey (CNHSS), which assesses labor market conditions in Brazil. Such surveys were conducted using a wide sample, including over 210,000 households distributed across approximately 3,500 municipalities (23). The CNHSS utilized updated concepts, definitions, and nomenclature in compliance with the International Labor Organization (ILO), established at the last International Conference of Labor Statisticians held in Geneva in 2023 (24). The main objective is to build a profile of different populations in relation to the job market, including active-age individuals, workforce participants, the employed, the unemployed, and those outside the workforce. For employed individuals, aspects such as occupational position, job category, employment level, and unemployment rate are recorded.

Employment level, which expresses the proportion of employed individuals in the population aged 14 years and older, was used to improve the comprehension of the geographic distribution of leptospirosis. This indicator varies among Brazilian states ranging nationwide between 49 and 58.9% over the years, reaching 57.6% by 2023 (25). This study assessed the CNHSS data for individuals aged 14 years and older, employed in the reference week, by occupational cluster in the main job in the fourth trimester of 2022 based on the Brazilian classification of household occupations. An age of 14 years was defined as the starting point for the labor age analysis based on the Brazilian legal and statistical framework. According to the Brazilian Federal Constitution (Article 7, XXXIII) and the Consolidation of Labor Laws, 14 years is the minimum age at which individuals can legally enter the workforce as apprentices (26). Furthermore, the Brazilian Institute of Geography and Statistics, the official data provider on the country’s workforce, has adopted the 14-and-older criterion for classifying the “Working-Age Population” in its national survey reports (27).

To ensure comparability, the 100 most populated cities (population > 274,000) were selected to guarantee minimum healthcare infrastructure capacity for leptospirosis attendance. To ensure the accuracy of the normalization model, occupational incidence rates were calculated using the Economically Active Population (EAP) of each municipality as the denominator. Categories that did not comprise the formal or active workforce, such as students, retirees, and homemakers, were excluded from this specific denominator to ensure that the calculated strictly reflected work-related exposure and professional statistical associations (27).

Population data were obtained from the BIGS using the latest nationwide census conducted in 2022. In the cities, confirmed cases of individuals aged 14 years and older, from 2007 to 2022, who self-reported their occupation, were classified into 10 major occupational clusters and three risk levels for leptospirosis (low, medium, and high), allowing for comparisons with the CNHSS dataset.

### 2.3 Occupational groups and risk classification

For comparative analyses, professional groups were initially categorized *a priori* into low-, medium-, and high-risk tiers based on their potential for direct exposure to *Leptospira* spp., ensuring the methodological independence and preventing circular reasoning. The classification was operationalized according to three pre-established criteria derived from the scientific literature and the epidemiological profile of leptospirosis in Brazil: (i) environmental exposure, including the frequency and intensity of contact with known risk factors, such as contaminated mud, surface water, sewage systems, garbage accumulation, or flood-prone environments; (ii) animal exposure, involving the handling of livestock, wildlife, or domestic animals in potentially contaminated settings; and (iii) occupational handling, related to the clinical management or laboratory processing of infected samples or individuals. This categorization functions as a theoretical and conceptual framework designed for comparative epidemiological risk assessment within the active workforce, rather than as a formally validated clinical tool.

### 2.4 Data selection and processing

**Figure 1:**
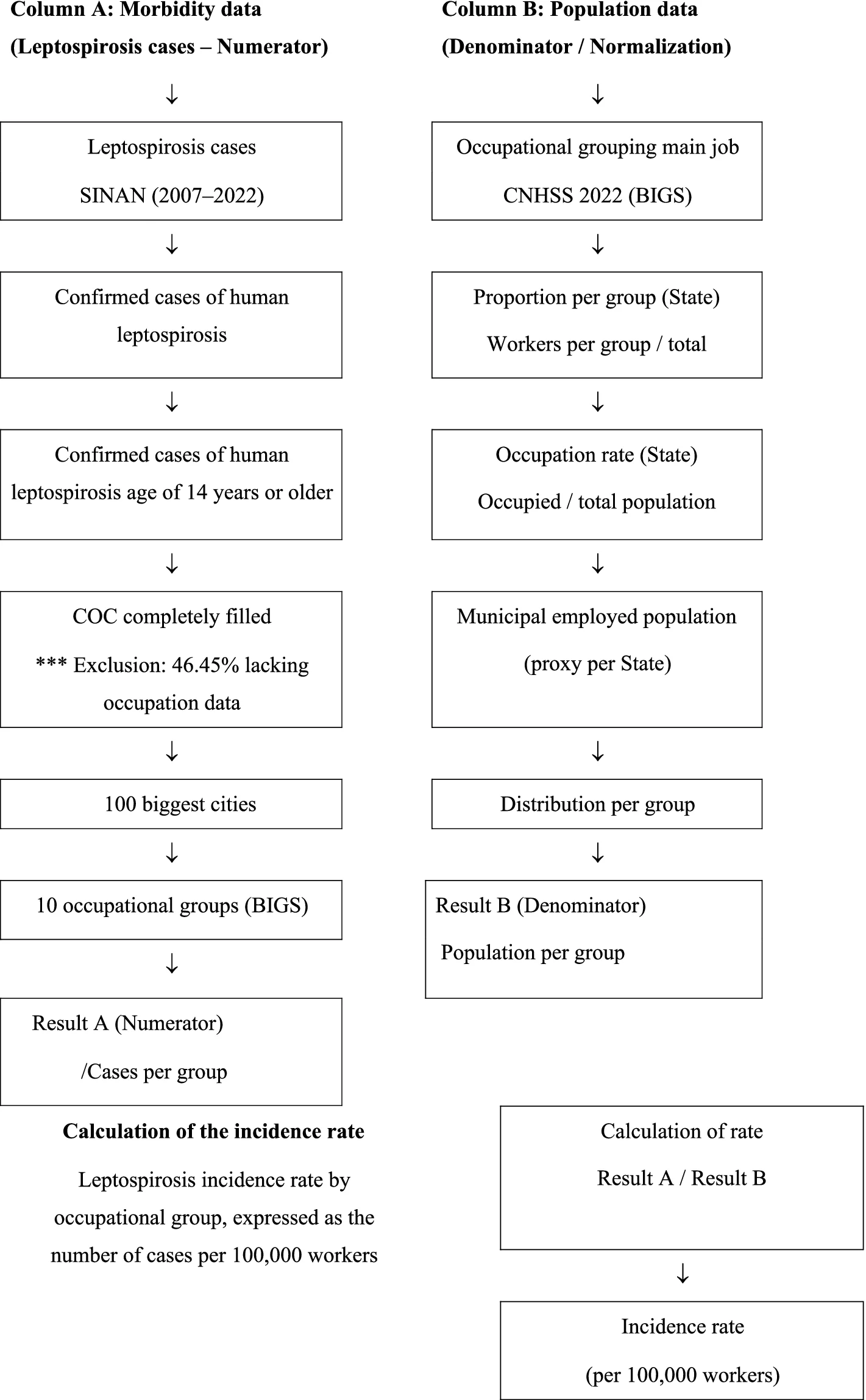
Flowchart of data selection and processing for the calculation of the standardized incidence rate of occupational leptospirosis. Column **A** shows the filtering steps applied to confirmed leptospirosis cases (numerator), while Column **B** describes the estimation of the employed population by occupational group (denominator). The convergence of both columns resulted in the incidence rate per occupational group, expressed as the number of cases per 100,000 workers.

The process of data selection and processing is summarized in the flowchart (Figure 1). Column A describes the filtering of leptospirosis cases reported to SINAN between 2007 and 2022, including only confirmed cases in individuals aged 14 years or older. Records were restricted to those with a completed occupational code (COC), which resulted in the exclusion of 46.45% of cases lacking this information. Subsequently, only residents of the 100 largest Brazilian municipalities were included, and cases were grouped into 10 major occupational categories according to the BIGS classification. This step produced the numerator of the incidence rate, i.e., the number of cases per occupational group in each municipality.

The Column B presents the estimation of the employed population by occupational group, derived from the Continuous National Household Sample Survey (CNHSS/BIGS, 4th quarter of 2022). For each state, the proportion of workers in each occupational group and the overall employment rate were calculated. These proportions were applied to the municipal populations, allowing the estimation of the number of workers per occupational group in each city. This step produced the denominator of the incidence rate. The convergence of the two columns enabled the calculation of the standardized incidence rate of leptospirosis by occupational group, expressed as the number of cases per 100,000 workers.

### 2.5 Occupational normalization for the 100 biggest Brazilian cities

The infection rate was calculated by dividing the number of cases in each occupational group by the estimated number of workers in that group in the 100 most populous Brazilian cities. This procedure was adopted to enable reasonable comparisons between cities and occupational categories, thus reducing potential biases caused by differences in workforce composition, population size, and reporting coverage.

The occupational structure across the 100 most populous Brazilian cities was estimated using data from the Continuous National Household Sample Survey (Continuous CNHSS), conducted by the BIGS. This survey collected quarterly information from approximately 211,000 households distributed across approximately 3,500 municipalities, ensuring representation of both formal and informal workers.

To derive the city-level occupational estimates, the state-level employment rate, which was adopted as a proxy, was used as an indicator. This procedure involved multiplying the employment rate of each state by the total resident population of the selected cities within that state. This allowed us to estimate the total number of employed people in each city.

Next, these totals were distributed among the 10 major occupational clusters defined by the Brazilian Classification of Occupations using the proportional distribution observed in the Continuous CNHSS, for each state. This approach ensured that the occupational composition of each city reflected broader state-level patterns, while maintaining internal consistency across cities. Finally, confirmed leptospirosis cases (2007–2022) were matched to their respective occupational clusters.

### 2.6 Statistical analysis

For descriptive analyses, all suspected and confirmed leptospirosis cases were included. The variables analyzed included year, city of notification and residence, age, sex, ethnicity, school level, occupation, exposure factors in the last 30 days, final classification of cases (laboratory or epidemiological), available data on potential places of infection, such as city, state, area (urban, peri-urban, or rural), environment (residence, work, pleasure, others), and case outcome. The exposure factors assessed included self-reported risk situations during the 30 days before clinical onset, as described in interviews with patients with suspected leptospirosis. These situations involve exposure to water or mud from flooding; water bodies such as rivers, creeks, lakes, ponds, or water reservoirs; livestock and agricultural activities; bean storage; rodent presence; direct contact with rodents; wastelands; garbage; water tanks; and sewage services.

To ensure notification capacity and data reliability, the analysis in the present study was restricted to the 100 largest Brazilian cities. Although such an approach may introduce urban bias, these cities are geographically distributed throughout the 26 Brazilian states and the federal district, functioning as regional referral centers for leptospirosis diagnosis and treatment.

Differences in incidence rates between Brazilian regions and occupational risk categories were verified using a Linear Mixed Model (LMM), implemented via the ‘lmer’ function from the *lme4* package (28) in R software (29). The LMM was preferred over generalized linear models, such as Poisson or Negative Binomial regression, because the response variable did not consist of raw discrete count data, but rather standardized continuous rates. These rates were calculated by dividing the number of cases by the working-age population (14 years and older) of each city and weighing the result by the respective state-level occupational proportions, to establish corrected weights for each category.

In this model, five Brazilian regions and three occupational risk levels (low, medium, and high) were included as fixed effects. To account for the temporal structure of the historical series (repeated measures from 2007 to 2022) and handle the unobserved local heterogeneity, the 100 cities were included as random effects (30). Statistical significance (*p* ≤ 0.05) was assessed using the ‘emmeans’ package (31), utilizing the ‘emmeans’ function to calculate estimated means and the ‘contrast’ function for paired comparisons. Model validation was systematically conducted through residual analysis, and visual diagnostics, including Q-Q plots and residual-versus-fitted value plots, were utilized to confirm that the assumptions of normality and homoscedasticity of the residuals were adequately met prior to assessing statistical significance.

## 3 Results

The Brazilian Ministry of Health reported 261,218 suspected and 55,779 (21.4%) confirmed cases of human leptospirosis between 2007 and 2022, of which 47,917 (85.91%) were confirmed by laboratory diagnosis and 7,183 (12.88%) by clinical epidemiological criteria. Of the confirmed cases, 960 (1.72%) ignored the patient’s occupation and 10,769 were officially considered occupational infections. Importantly, occupation was not recorded in 46.45% of the cases, a gap likely related to the manual completion of forms by healthcare professionals and the lack of systematic data qualification (Supplementary Figure 1). To evaluate potential notification bias, we compared demographic and regional characteristics of cases with complete occupational data (*n* = 29,868) and those with missing information (*n* = 25,911). As shown in Supplementary Table 1, both groups presented similar distributions, with a predominance of males and individuals aged 20–39 years, although the southern region accounted for proportionally more cases with complete occupational data. These findings suggest that, while missingness may not be entirely random, the overall demographic and geographic profiles remain consistent across groups.

Among the 27,998 confirmed human leptospirosis cases at labor age (14 years or older) from 2007 to 2022 in Brazil, the 10 most frequent occupations included students (10.6%), agriculture-livestock workers (8.1%), construction workers (7.6%), housekeepers (7.4%), retired persons (6.1%), chronically unemployed persons (4.2%), recycling material collectors (2.5%), agriculture temporary workers (2.1%), agriculture polyvalent workers (2.0%), and construction contractors (1.8%; Supplementary Table 2).

Among confirmed leptospirosis cases in individuals aged ≥14 years, the analysis of clinical severity by occupational groups showed higher frequencies of renal failure, jaundice, and pulmonary hemorrhage among agricultural, construction, and waste collection workers. These groups also presented higher hospitalization and case fatality rates, as detailed in Supplementary Tables 7A,B.

**Figure 2:**
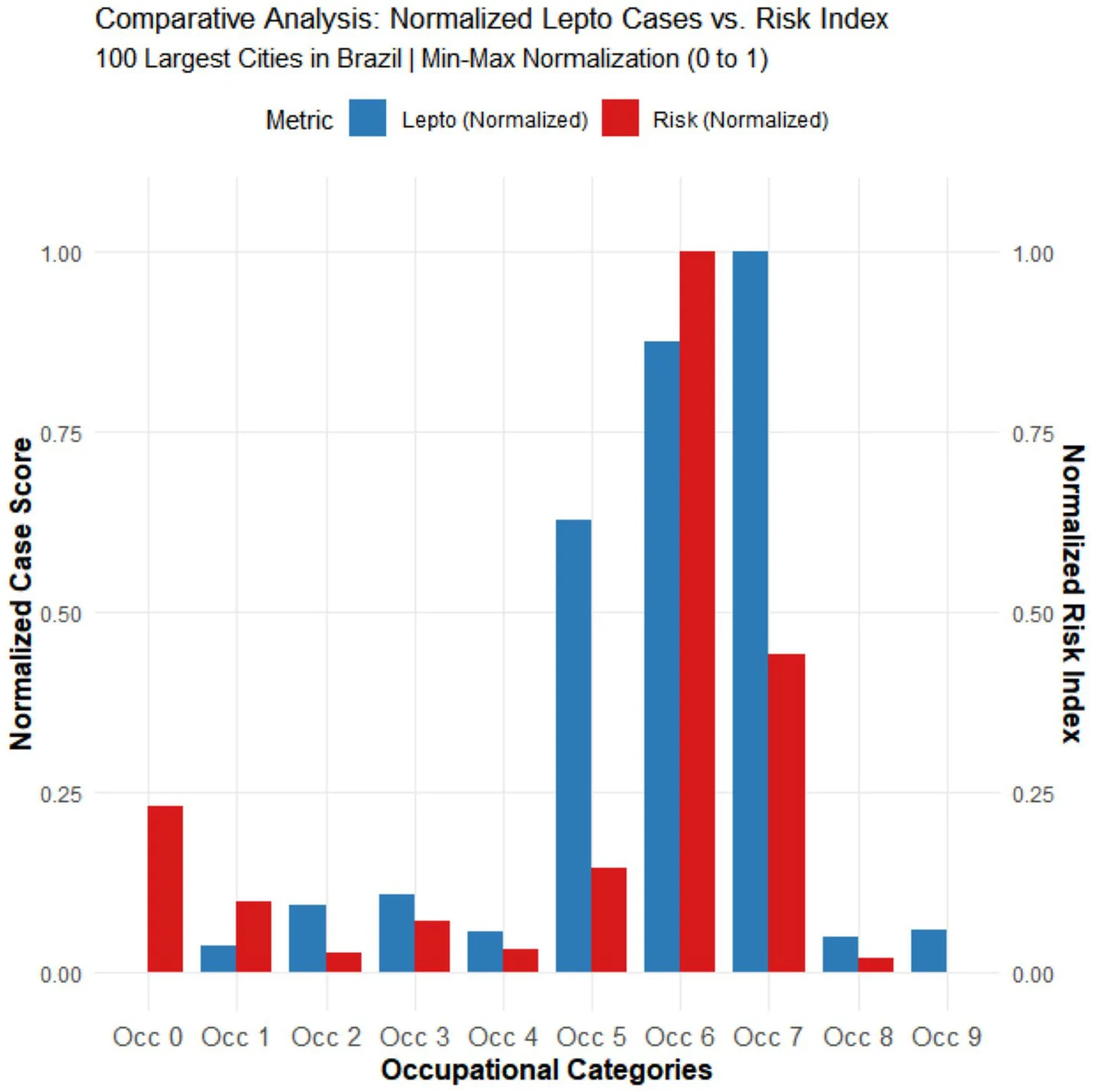
Comparative analysis between the absolute volume of cases and the normalized occupational risk. Blue bars represent the scaled count of occurrences by occupation, while red bars indicate the normalized risk rate relative to the labor force size. Data were proportionally scaled using Min-Max normalization (0 to 1) to allow direct visual comparison between case magnitude and risk intensity. Occupational categories are defined as follows: Occ 0: Armed Forces, Police, and Firefighters; Occ 1: Senior Officials and Managers; Occ 2: Science and Art Professionals; Occ 3: Mid-level Technicians; Occ 4: Administrative Workers; Occ 5: Service and Sales Workers; Occ 6: Agricultural, Forestry, and Fishing Workers; Occ 7: Industrial Production Workers (Craft and Related Trades Workers); Occ 8: Industrial Production Workers (Plant and Machine Operators and Assemblers); Occ 9: Repair and Maintenance Workers.

Among the 10,769 leptospirosis cases confirmed as occupational infections, the 10 most frequent occupations included agricultural workers in general (12.0%), construction workers (5.8%), recyclable material collectors (4.6%), agricultural polyvalent workers (2.7%), flying agricultural workers (2.6%), students (2.0%), rice cultivation workers (1.8%), construction workers (1.4%), housekeeper agricultural (1.3%), and retired persons (1.3%; Supplementary Table 3). The patients were mostly men (9,863; 91.6%), with an average age of 31.5 years. The environment identified as a potential source of infection was mostly the workplace (8,126; 75.46%), followed by households (1,617; 15.02%), pleasure (50; 0.46%), others (169; 1.57%), while the environment was ignored in 219 (2.03%) cases and 588 (5.46%) did not answer. The confirmed cases were mostly from urban (4,797; 44.54%) infection areas, followed by rural (4,776; 44.35%), peri-urban (410; 3.81%), with 163 (1.51%) ignored cases, and 623 (5.79%) without information (Figure 2).

Based on the frequency and intensity of exposure and potential contact with infected people or animals, 10 major occupational clusters were identified (Table 1).

**Table 1:** Occupational clusters categorized by leptospirosis risk level and pairwise statistical significance.

Risk level	Occupational clusters	Statistical significance (Pairwise Contrast)
Low	1. Directors and managers 4. Administrative support workers	Reference group
Average (Medium)	2. Science and health professionals (including veterinarians) 3. Technicians and mid-level professionals 8. Machine operators and assemblers 9. Elementary occupations 0. Armed forces, Police, and Firefighters	*p* = 0.9992 (vs. Low risk)
High	5. Cleaning and street workers (garbage collectors and recyclers) 6. Skilled workers in livestock, forestry, and fisheries 7. Skilled workers in construction, mechanics, and mining	*p* < 0.0001 (vs. Average risk) *p* < 0.0001 (vs. Low risk)

**Figure 3:**
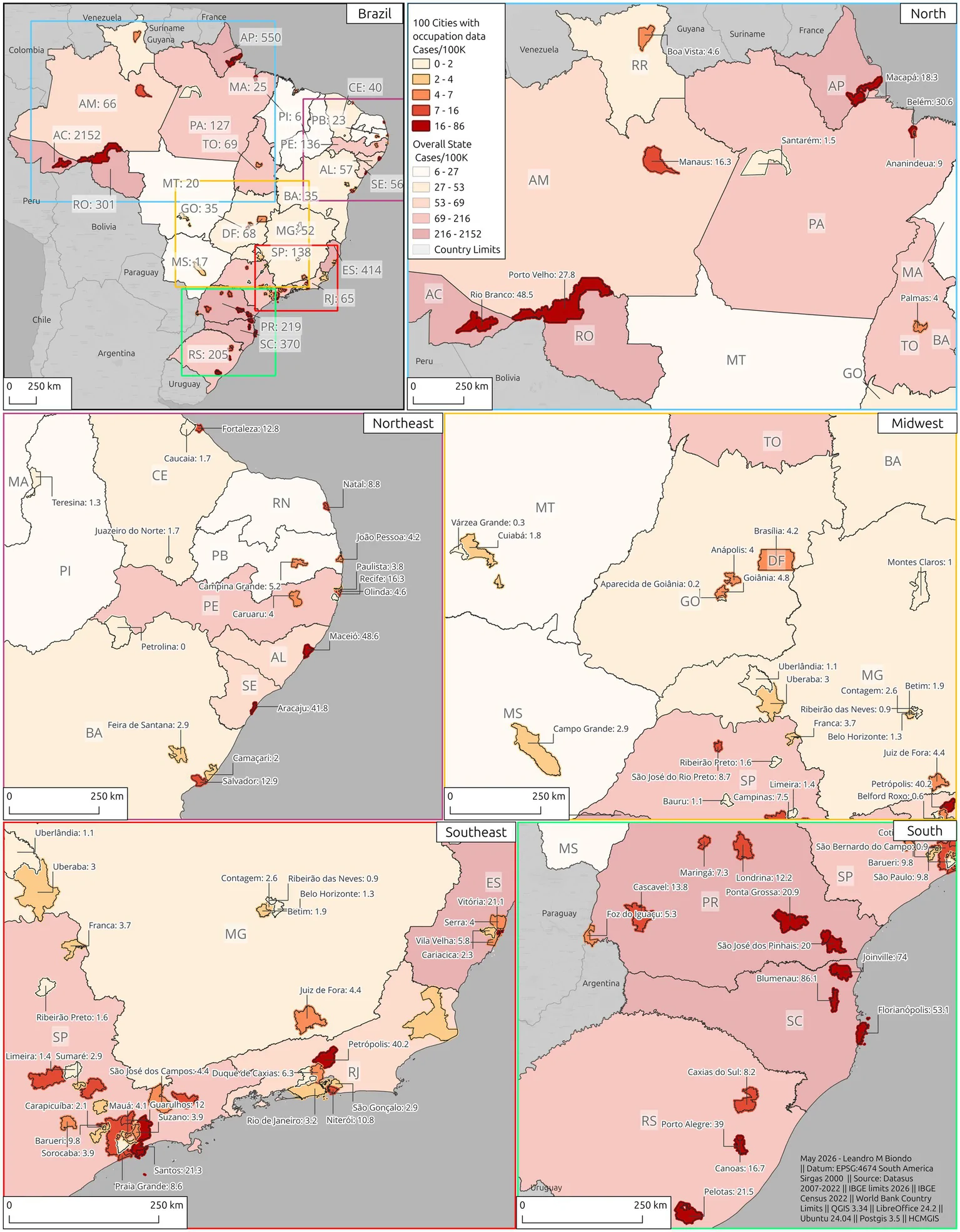
Brazilian leptospirosis cases per 100,000 inhabitants in each state and the detailed regions with the 100 studied city limits highlighted. The cities are colored by the cases/100 K inhabitants with available data on occupation. Map made by the authors with municipalities and state limits from BIGS, BC250 2025 geospatial data (60), country limits from World Bank (61), population estimates from BIGS, 2022 census (17), and background rendered with HCMGIS (62) and QGIS (63).

The analysis of comparative leptospirosis prevalence standardized incidence rates was based on the 100 largest Brazilian cities to ensure equal attendance and notification (Figure 3), including the assessment of the resident population and employed labor force per Brazilian region, according to the Brazilian Institute of Geography and Statistics (2022; Supplementary Table 2), and persons in labor aged 14 years or over, employed in the reference week, by occupational group of the main job, Brazil, and Greater Region (Supplementary Table 3).

The southern region had the highest occurrence of leptospirosis in Brazil, as represented by significant differences when compared with the northeastern, southeastern, and central-western regions, but not with the northern and northeastern regions (Supplementary Table 4). Occupational groups classified as high risk for leptospirosis in Brazil had a significantly higher occurrence than the medium- and low-risk groups.

**Figure 4:**
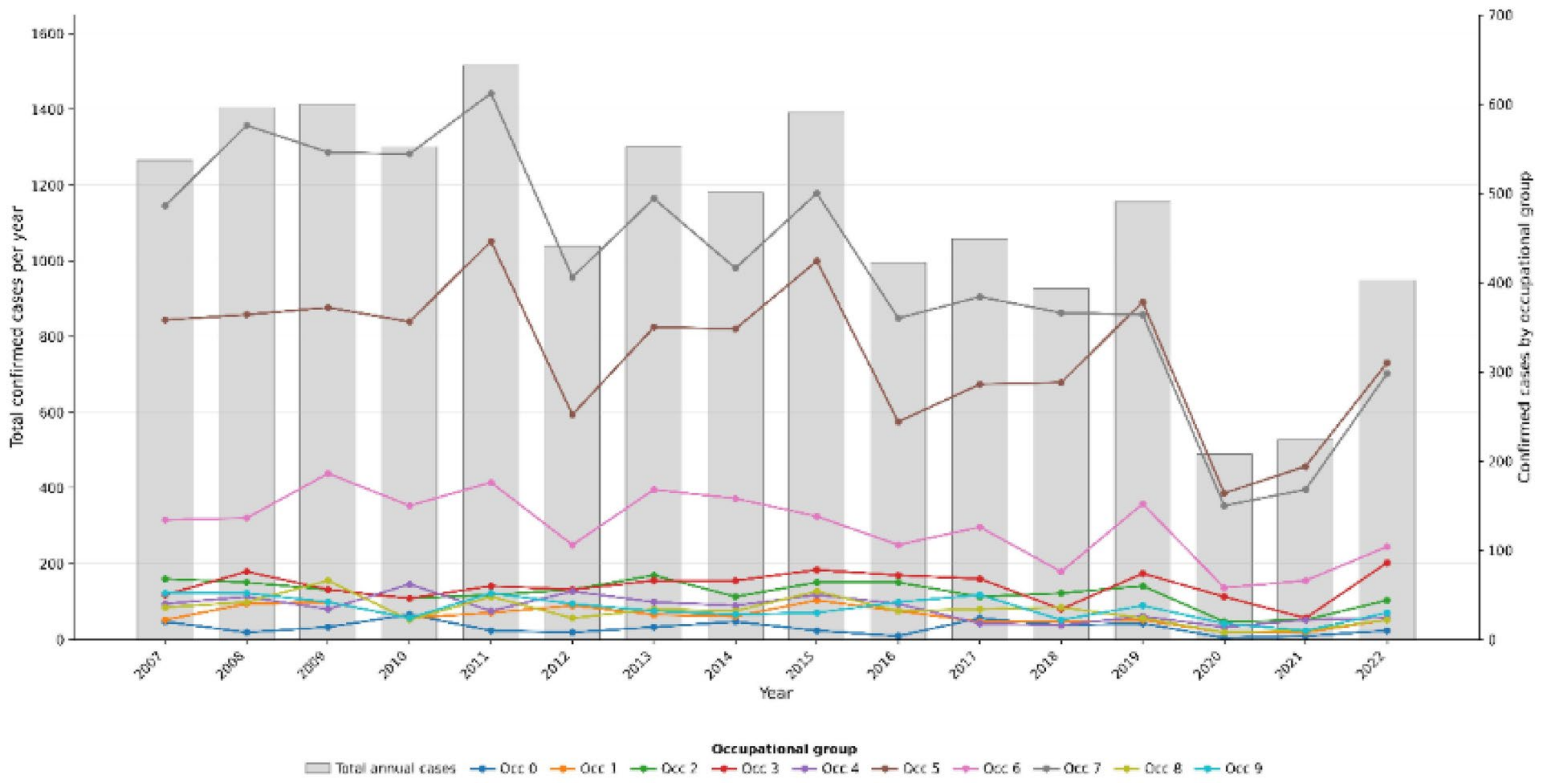
Temporal evolution of confirmed occupational leptospirosis cases from 2007 to 2022. Bars represent the total number of confirmed cases per year, shown on the left y-axis, while lines represent the annual number of confirmed cases for each occupational group, shown on the right y-axis. Occupational groups are defined as follows: Occ 0, Armed Forces, Police, and Firefighters; Occ 1, Senior Officials and Managers; Occ 2, Science and Art Professionals; Occ 3, Mid-level Technicians; Occ 4, Administrative Workers; Occ 5, Service and Sales Workers; Occ 6, Agricultural, Forestry, and Fishing Workers; Occ 7, Craft and Related Trades Workers; Occ 8, Plant and Machine Operators and Assemblers; and Occ 9, Repair and Maintenance Workers.

The temporal distribution of confirmed occupational leptospirosis cases from 2007 to 2022 is shown in Figure 4. Bars represent the total annual number of confirmed cases, while lines show the annual variation in the number of cases within each occupational group. Overall, the temporal pattern indicates fluctuations over the study period, with higher annual totals observed in selected years and a marked reduction in 2020 and 2021, followed by an increase in 2022.

## 4 Discussion

To the best of our knowledge, this is the first study to assess leptospirosis cases gathered by SINAN using data normalized to censitary working occupations per Brazilian region. The classification of low-, medium-, and high-risk groups based on the frequency, intensity of exposure, and potential contact with infected people or animals has highlighted the differences in leptospirosis risk according to the type of activity performed. Beyond allowing a stratified analysis of notified cases by occupational profile, this division also provides a framework for interpreting the findings obtained from the ranking of professions with the highest number of cases. This approach facilitates the identification of the most vulnerable groups and helps understand how the exposure factors described in the literature are reflected in the observed epidemiological profile.

Based on the frequency, intensity of exposure, and potential contact with infected people or animals, clusters with a high risk of exposure were composed of street cleaners (garbage collectors and recyclers) and street sellers; skilled workers in livestock, forestry, and fishery; and skilled workers in construction, mechanics, and mining. Garbage collection is also recognized as a highly exposed occupation in Malaysia and Paraguay (10, 32). Garbage collectors may experience prolonged exposure to contaminated garbage, soil, and water. Additionally, poor knowledge of the cause, mode of transmission, symptoms, and treatment of leptospirosis may hinder diagnosis (33, 34).

A previous systematic review has analyzed studies from 2012 to 2021 and shown slaughterhouse workers presented the highest risk of infection (OR 5.1–7.5), along with forestry workers (AOR 9.2), military (AOR 26.65), urban sweepers (OR 2.29), construction workers (AOR 3.76), and farmers (AOR 1.6) (9). This study found that a higher occupational exposure to *Leptospira* spp. was associated with contact with contaminated water, soil, and infected animals. Additionally, military training and on-duty assignments, usually related to skin injury, may predispose individuals to infections (9).

In the present study (Supplementary Table 2), the occupations with the highest frequency included farmers (12.0%), construction workers (5.8%), and recycling material collectors (4.6%), with the work environment identified as the most common site of infection (75.5%). These occupational patterns, however, must be understood within a broader environmental and infrastructural context. Recent spatial epidemiological research indicates that leptospirosis risk is associated with proximity to drainage systems, soil impermeability, and social vulnerability, highlighting how environmental and sociostructural conditions shape exposure patterns (35). For construction and urban workers, exposure often occurs through contaminated garbage, soil, and water in areas with precarious sanitation (35, 36). Similarly, recycling material collectors’ exposure is linked to contact with contaminated waste without adequate personal protective equipment (PPE), often in territories characterized by high social vulnerability and environmental neglect (24). Therefore, the observed occupational patterns are inextricably linked to underlying socio-spatial determinants, where professional activity overlaps with high-risk environmental landscapes.

Although our statistical model categorized military personnel and firefighters within the medium-risk group, it is crucial to recognize that their risk profiles are highly episodic and context-dependent. During natural disasters such as the major floods frequently observed in Brazil, these professionals act as first responders, directly interacting with contaminated water and mud in uncontrolled environments. This operational reality exponentially increases their exposure to *Leptospira* spp., a risk well-documented in the literature concerning rescue teams in flood-stricken regions (37). Furthermore, military forces often undergo training or missions in peri-urban and jungle areas, where the environmental load of the pathogen is high, leading to reported outbreaks even in high-performance troops (38). Therefore, the ‘medium-risk’ classification should be viewed as a baseline that does not account for acute peaks of exposure during emergency mandates, justifying the need for specific PPE protocols and post-exposure monitoring for these categories (36).

- A previous study on slaughterhouse and meat workers revealed 16/250 (6.4%) seropositive individuals in South Sudan (35), 10/39 (25.6%) in India (39), 28/172 (16.28%) in Burkina Faso (40), 8/9 (88%) in Mexico (41), 99/737 (13.4%) in Kenya (42), 49/592 (8.3%) in New Zealand (43), 2/102 (1.96%) in Turkey (44), and 6/150 (4%) in Brazil (45). Despite being highly exposed, such professional groups have also accounted for low absolute numbers compared with other exposed occupations.

The present study has shown that the most exposed working groups, in general, were low-income professionals engaged in hand-labor work, leading to direct contact with contaminated water and soil. Although such occupational contact may occur in other professions such as marine mammal rescue and rehabilitation workers, with 2/213 (1%) seropositive workers in California, USA, preventive deficiencies may be modified and resolved through improvements in handling and other safety protocols (46). In addition, despite potentially higher exposure, such groups may account for a low absolute number of professionals among professions with the highest exposure compared to fishery workers. In the present study, freshwater fishermen were ranked as 46th of the most affected occupations with 88 confirmed cases, followed by professional sea fishermen (48th) with 83, artisanal fishermen/shrimp (78th) with 44, fish cleaning (fishery) workers (350th) with 4, industrial fishermen (507th) with 2, and fish cook (816th) with 1 confirmed case; lobster catchers and fishery engineers had no cases; veterinarians (74th) presented 48 and biologists (213th) 8 confirmed cases.

Considering the geographic distribution, the southern region presented the highest normalized occurrence of the disease, in agreement with the epidemiological situation provided by the Brazilian Ministry of Health, which indicates that most cases were concentrated in the southern and southeastern regions (47). This spatial pattern is corroborated by previous temporal analyses showing persistently high prevalence and hospitalization rates in these two regions over time (48). While it could be hypothesized that the southern climate plays a role, it is critical to note that Brazil is mostly located within the intertropical zone (~92%), presenting equatorial, tropical, and subtropical climates rather than a temperate climate. Therefore, this higher notification rate is likely a consequence of a superior efficiency of the local diagnosis and surveillance system (SINAN), along with better overall access to healthcare services in these regions (49). Additionally, these highly populated urban centers feature advanced urbanization where dense populations overlap with synanthropic rat reservoirs, facilitating disease transmission (48). Given this scenario, tropical rainfall combined with inadequate urban drainage triggers critical exposure; thus, future data surveys must systematically consider extreme climate events such as major floods and droughts to fully analyze and establish the occurrence and distribution of occupational leptospirosis (50).

Another study also showed a temporal analysis of leptospirosis during 2011, 2015, 2019, and 2021 and reported the highest prevalence and hospitalization in the southern and southeastern regions over the years, whereas the midwestern and northern regions showed the lowest numbers (48). This study hypothesized that the southern and southeastern Brazilian regions present higher urbanization and that most populated areas overlap with rat populations, facilitating disease transmission (48). In addition, the higher prevalence and hospitalization in the southern and southeastern regions could be a consequence of the better efficiency of the local diagnosis and surveillance system, along with better access to health services (49).

In urban areas, human *Leptospira* infection may be facilitated by high human population density and precarious infrastructure conditions, including low sanitation, non-potable and unsafe water sources, garbage accumulation, and inefficient rat control and prevention, leading to a high rodent presence as *Leptospira* spp. infection sources (4). Trash collectors may be at high occupational risk due to direct contact with rats, rat urine, and contaminated trash (4). In addition, flooding caused by heavy rainy seasons and natural disasters such as rainstorms and hurricanes may lead to leptospirosis outbreaks (36). At lower frequencies, infection may occur by direct contact with the blood, tissues, and organs of infected animals, accidental transmission in laboratories, and ingestion of contaminated water and food; human-to-human transmission has rarely been reported (51).

Out of the 27,998 human confirmed leptospirosis cases at labor age (14 years or older) from 2007 to 2022 in Brazil, the 10 most frequent occupations included students (10.6%), agriculture-livestock workers (8.1%), construction workers (7.6%), housekeepers (7.4%), retired persons (6.1%), chronically unemployed persons (4.2%), recycling material collectors (2.5%), agriculture temporary workers (2.1%), agriculture polyvalent workers (2.0%), and construction contractors (1.8%). Regarding the descriptive occupational rankings (Supplementary Table 2), it is important to note that students and retirees had high absolute frequencies. However, these categories were excluded from the normalization models. In Brazil, these groups represent massive demographic contingents; according to the latest national data, there are over 48 million students enrolled in basic and higher education (17, 27) and more than 30 million retirees and pensioners (27).

Including these non-active populations in the denominator of occupational risk would introduce a significant dilution bias, artificially lowering the incidence rates of active professions and masking the specific association between labor activities and *Leptospira* spp. exposure. Therefore, their exclusion was a methodological requirement to ensure that the calculated risk strictly reflected the exposure patterns within the economically active workforce. Although students represented 10.6% of the reported cases in working-age individuals, their prominence in the absolute data suggests the occurrence of non-work-related exposures, such as leisure activities or peridomestic risks, commonly observed among young urban adults. Additionally, this high frequency in the numerator reflects the reality of SINAN notification forms in which young individuals are frequently classified as students because of a lack of formal employment. This distinction reinforces the importance of data normalization. While absolute numbers pointed to students as a frequent group, the normalized rate successfully revealed where the risk was truly linked to the productive sector. As the highest ranked in absolute numbers of confirmed leptospirosis cases, these occupations may be considered the most important for healthcare attendance and capacity, which may be overwhelmed, saturated, and collapsed during leptospirosis outbreaks and epidemics.

As expected, the high-risk working groups for leptospirosis in Brazil showed statistically higher differences than the medium- and low-risk groups. The confirmed cases were approximately 80% men, mostly of white and brown ethnicities, between 25 and 29 years of age, with over half self-declared illiteracy or an incomplete basic educational level. Previous studies in Brazil have shown similar results, with a survey from 2010 to 2015 showing 79.4% men, 68.3% from 25 to 59 years old, 53.4% of white ethnicity, and 51.1% illiterate or with incomplete basic educational levels (22).

The results obtained in this study corroborate those of other retrospective studies, which found the highest prevalence in Caucasians (45.0%) and men (79.5%), mostly between 20 and 39 years old (39.5%), and with an incomplete basic school level (15.8%) (52). The data from to 2020–2015 showed that it mostly affected 15–59-year-old adult men, impacting workforce ages, with a slightly higher median age in rural areas and a male-to-female ratio of 4:1 (22). In addition, white people had a higher incidence of leptospirosis in both rural and urban areas, whereas indigenous populations were more affected by the disease in the central-western and northern Brazilian regions, with a higher overall presence of indigenous populations (22). Another survey of the SINAN reports from 2007 to 2015 also showed a higher prevalence in men (78.6%) and white people (46.0%), followed by black and brown (41.2%) ethnicities, and mostly between 20 and 59 years old (72.3%) with an incomplete basic school level (35.6%) (53).

A survey of American countries revealed that leptospirosis cases were mostly male, with a median of 66.7% (40.5–90.9%) (2). In rural areas, this study found that agricultural workers, particularly rice and sugarcane workers, were more likely to be infected, mainly because of contact with contaminated water and soil during labor, in addition to the higher risk associated with rainstorms and flooding. Urban areas are mostly affected in slums and low-income neighborhoods, with precarious sanitary infrastructure and open sewers contributing to rats (main leptospirosis reservoirs) and disease dissemination, in addition to rainfall and flooding (2). This predisposition has been observed in Brazil, where the incidence is higher among agricultural workers in rural areas and slums in urban areas. In other South American countries, including Peru, Colombia, Ecuador, and Costa Rica, incidence patterns vary according to predominant local activities such as agriculture, livestock, and indigenous community routines. Nonetheless, the epidemiology of leptospirosis in each country is unclear due to underreporting and neglect (2).

A retrospective (2000–2015) epidemiological survey of leptospirosis in Brazil showed distinct incidence patterns, risk factors, and preventive measures throughout the country (22). As observed here, a higher incidence was observed in men, of white ethnicity, and in the southern region. Another study reported that most confirmed cases were from urban areas (83.1%) in the southern and southeastern regions (22). This study indicated that agricultural workers, particularly rice and livestock workers, are most exposed to the disease in rural settings owing to their direct contact with water and soil contaminated by rodent urine. In urban areas, construction workers, particularly those in early buildings, are at a higher risk of exposure to contaminated water and soil. Other risk groups include miners, trash and recycling collectors, and inmates because work conditions and precarious households may predispose them to rodent proliferation and leptospirosis transmission (22).

Another study focused on the spatial and temporal distribution of leptospirosis between 2007 and 2017 and found 42,310 cases, with an annual average of 3,846 cases and a prevalence of 1.9/100,000 inhabitants (6). Overall, 2,600 of 5,570 (46.67%) cities had confirmed cases, mostly in southern and northern Brazil. Despite having the highest absolute number of cases, the southeastern region (where the cities of São Paulo and Rio de Janeiro are located) had below the national average of cases after per capita normalization (6).

The biological plausibility of the occupational risk patterns identified is reinforced by the distribution of severe clinical outcomes across professional groups. As shown in Supplementary Tables 7A,B, occupations with higher exposure loads presented greater frequencies of renal failure, jaundice, and pulmonary hemorrhage, as well as higher hospitalization and case fatality rates. These findings suggest that intense and repeated contact with contaminated environments predisposes workers to more severe clinical forms of leptospirosis, increasing the burden on health services and amplifying the risk of death. Classic studies had already described acute kidney injury and jaundice as frequent manifestations in hospitalized patients (54) as well as pulmonary hemorrhage as a marker of severity in rural workers (55, 56). Comprehensive reviews and recent studies consolidate these findings (14) (57–59), corroborating our results and supporting the need to integrate occupational health surveillance with clinical monitoring.

A limitation of the present study is the high proportion of missing information in the notification forms. In suspected cases, 49.52% lacked occupational data, and similar gaps were observed for other variables such as ethnicity (12%) and education level (35%) (11), which prevent adequate analyses of associated risk factors. Leptospirosis disproportionately affects low-income and low-educated individuals with limited access to healthcare, leading to underdiagnosis and underreporting. This invisibility is exacerbated by weaknesses in the SINAN reporting system, which should function as a crucial tool for notification and feedback (11).

In confirmed cases, 46.45% lacked occupational information, representing a significant limitation for calculating incidence rates. Nevertheless, the use of a 16-year historical series (2007–2022) provided a large sample of over 29,000 entries with complete occupational data. As shown in Supplementary Table 1, the demographic and regional distributions of cases with missing data were broadly similar to those with complete records, with predominance of males and individuals aged 20–39 years across both groups. These findings suggest that the occupational risk patterns reported here pertain to the subset of cases with complete occupational data and should be interpreted in that light. Although absolute rates may have been underestimated, the identified risk hierarchy and trends remain epidemiologically consistent. Importantly, the high proportion of missing occupational information highlights the need to strengthen data quality in the Brazilian surveillance system (SINAN), ensuring systematic and standardized reporting of occupational variables. Improved notification practices would enhance the accuracy of future analyses and support more effective public health interventions targeting occupational risk groups.

The temporal analysis (Figure 4) demonstrates that the occupational distribution of leptospirosis cases remained relatively stable over time, despite fluctuations in the total number of cases. This stability supports the robustness of the occupational risk patterns identified and suggests that the associations are not random artifacts of specific years. Overall, the temporal pattern indicates fluctuations over the study period, with higher annual totals observed in selected years and a marked reduction in 2020 and 2021, followed by an increase in 2022. These findings highlight that, although the incidence of leptospirosis varied across years, the occupational risk groups remained consistently represented, reinforcing the strength of the associations observed.

Another limitation is that although focusing on the 100 largest Brazilian cities has ensured data robustness and reporting capacity, this methodology may have introduced an urban bias. It may have led to an underestimation of the real risk in predominantly rural and agricultural professions, which may have been more prevalent in the smaller municipalities that were excluded from the analysis. Consequently, the results presented here more accurately reflect the leptospirosis scenario in urban and peri-urban contexts, where sectors such as construction and recycling have emerged as critical risk groups. Future studies should focus on laborers in agricultural and rural areas to fully establish the risk of leptospirosis in these occupational scenarios.

Furthermore, using state-level employment data from the Continuous National Household Sample Survey as a proxy for municipal occupational structures introduces uncertainties and potential ecological biases. This strategy assumes a degree of homogeneity in the workforce distribution within a state, which may not fully reflect local municipal realities. However, this approach was adopted as a necessary approximation for comparative purposes given the absence of updated annual census data detailing major occupational groups at the municipal level for the studied period. To mitigate the impact of this uncertainty, our analysis focused on the risk hierarchy and long-term trends, rather than on isolated absolute values. Thus, while the denominators used for risk estimation represent statistical approximations, they provide a reliable and standardized basis for valid comparisons between different economic sectors across large urban centers.

Finally, regarding the occupational risk tiers, we acknowledge the absence of a formal clinical validation of the low-, medium-, and high-risk classifications used in this study. Although this stratification was defined *a priori* based on robust literature concerning transmission pathways, such as environmental, animal, and occupational exposure, to ensure methodological independence and prevent circular reasoning, it lacks empirical validation. Therefore, it should be interpreted as a conceptual and theoretical framework tailored for comparative epidemiological assessment within the active workforce rather than as an absolute clinical indicator of individual risk.

Although the completion of occupational variables remains a challenge, dynamic monitoring enabled by SINAN (National System of Notifiable Diseases) has established the most strategic tool for addressing notification gaps in Brazil. The broad nationwide reach and capacity for the real-time integration of environmental and epidemiological data have allowed SINAN to function as a critical sensor for identifying failures in occupational health surveillance. This robust structure has transformed administrative records into health intelligence, which is crucial for guiding public policy and efficient interventions. Thus, the systematic use of this database may not only provide the identification of emerging risks, but also the opportunity to continuously correct and improve the quality of feedback information. Therefore, this study proposes the use of this national database to assess and analyze exposure patterns and adverse events related to occupational risk, to strengthen strategies for the prevention and promotion of workplace health. The present study showed that occupation is a predictor for leptospirosis, which varies in different regions according to the professional frequency. The SINAN may be used as an effective indicator of public health actions and policies, in addition to the dynamic monitoring of occupational exposure to leptospirosis over time. Finally, occupation should always be analyzed as a predictor after the normalization of professional frequency to ensure access to healthcare assistance and systematic notification.

## Data Availability

The original contributions presented in the study are included in the article/Supplementary material, further inquiries can be directed to the corresponding author.
