# Supplementary material for: Occupation as an associated risk factor for leptospirosis
Source: Front Public Health. 2026 Aug 17;14:1810977. doi: 10.3389/fpubh.2026.1810977 (PMC13526960; doi:10.3389/fpubh.2026.1810977)
Supplement: Supplementary file 1 [file Data_Sheet_1.pdf]

Supplementary Material

Supplementary Figure 1. Suspicious and confirmed leptospirosis cases,in occupational age and infection from 2007 to 2022 in Brazil, based on demographic data provided by the National Surveillance System, Brazilian Ministry of Health.

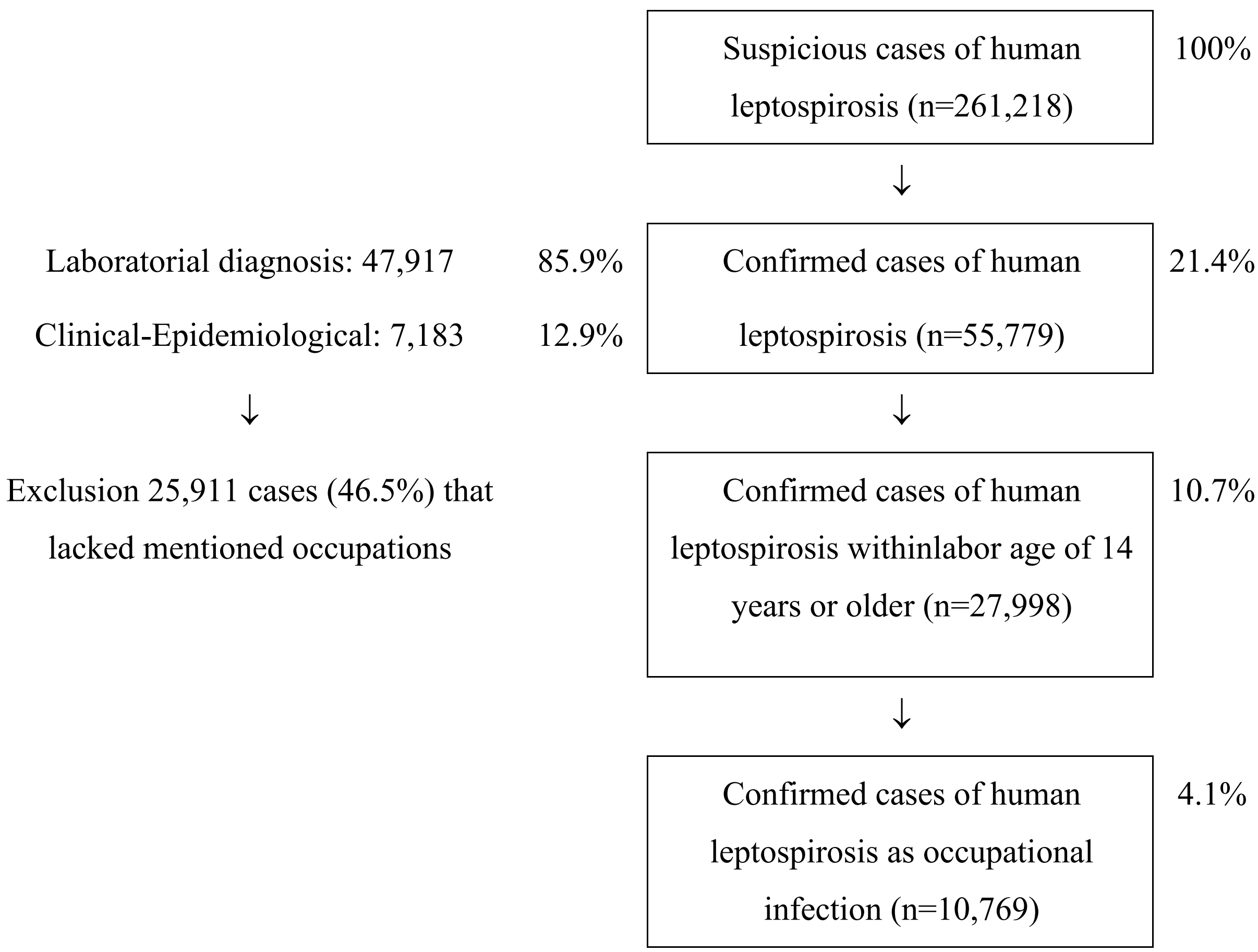

Supplementary Table 1. Comparative demographic and regional characteristics of confirmed leptospirosis cases in Brazil (2007–2022) with complete versus missing occupational information, including all cases and those restricted to the 100 largest cities.

Variable	All Brazil – Missing Occupation<br>n=25,911 (46.5%)	All Brazil – Complete Occupation<br>n=29,868 (53.5%)	100 Largest Cities – Missing Occupation<br>n=16,419 (51.7%)	100 Largest Cities – Complete Occupation<br>n=15,363 (48.3%)
Sex	Male: 20,555 (79.3%)<br>Female: 5,352 (20.7%)	Male: 24,147 (80.9%)<br>Female: 5,721 (19.1%)	Male: 12,836 (78.2%)<br>Female: 3,582 (21.8%)	Male: 12,319 (80.2%)<br>Female: 3,044 (19.8%)
Age group	<14: 2,173 (8.4%)<br>14–19: 2,446 (9.4%)<br>20–39: 10,723 (41.4%)<br>40–59: 8,245 (31.8%)<br>60+: 2,324 (9.0%)	<14: 1,870 (6.3%)<br>14–19: 3,475 (11.6%)<br>20–39: 11,653 (39.0%)<br>40–59: 9,960 (33.4%)<br>60+: 2,910 (9.7%)	<14: 1,534 (9.3%)<br>14–19: 1,627 (9.9%)<br>20–39: 6,838 (41.6%)<br>40–59: 5,000 (30.4%)<br>60+: 1,420 (8.7%)	<14: 968 (6.3%)<br>14–19: 1,877 (12.2%)<br>20–39: 6,040 (39.3%)<br>40–59: 4,991 (32.5%)<br>60+: 1,487 (9.7%)
Region	North: 5,329 (20.6%)<br>Northeast: 5,286 (20.4%)<br>Southeast: 9,443 (36.4%)<br>South: 5,497 (21.2%)<br>Center-West: 356 (1.4%)	North: 3,754 (12.6%)<br>Northeast: 3,885 (13.0%)<br>Southeast: 9,073 (30.4%)<br>South: 12,619 (42.3%)<br>Center-West: 537 (1.8%)	North: 4,179 (25.5%)<br>Northeast: 4,110 (25.0%)<br>Southeast: 6,180 (37.6%)<br>South: 1,664 (10.1%)<br>Center-West: 286 (1.7%)	North: 2,524 (16.4%)<br>Northeast: 3,095 (20.1%)<br>Southeast: 5,077 (33.0%)<br>South: 4,305 (28.0%)<br>Center-West: 362 (2.4%)

Supplementary Table 2. The ten most frequent occupations in the 27,998 human confirmed leptospirosis cases due to occupation at labor age (14 years or older) from 2007 to 2022 in Brazil, based on general demographic data provided by the National Surveillance System, Brazilian Ministry of Health by occupational group in the main job.

Occupation	Cases	%	Main Groups
1. Student	2,976	10.6	Outside the labour force (Non-economically active population)
2. Agricultural worker in general	2,255	8.1	Agricultural, forestry, hunting and fishing workers **(Skilled Agricultural, Forestry and Fishery Workers)**
3. Construction worker	2,116	7.6	Workers in the production of industrial goods and services **(Craft and Related Trades Workers)**
4. Housekeepers	2,084	7.4	Outside the labour force (Non-economically active population)
5. Retired person	1,697	6.1	Outside the labour force (Non-economically active population)
6. Chronic unemployed	1,186	4.2	Outside the labour force (Non-economically active population)

7.	Recyclable material collector	712	2.5	Service workers, salespeople in stores and markets**(Craft and Related Trades Workers)**
8.	Flying agricultural worker	596	2.1	Agricultural, forestry, hunting and fishing workers **(Skilled Agricultural, Forestry and Fishery Workers)**
9.	Multi-purpose agricultural producer	551	2.0	Agricultural, forestry, hunting and fishing workers **(Skilled Agricultural, Forestry and Fishery Workers)**
10.	Construction contractor	490	1.8	Workers in the production of industrial goods and services **(Craft and Related Trades Workers)**
	Others	13,335	47.6	
	Total	27,998	100	

Supplementary Table 3. The ten most frequent occupations in the 10,769 human confirmed leptospirosis as occupational infection cases due to occupation at labor age (14 years or older) from 2007 to 2022 in Brazil, based on general demographic data provided by the National Surveillance System, Brazilian Ministry of Health by occupational group in the main job.

	Occupation	Cases	%	Main Groups
1.	Agricultural worker in general	1,297	12.0	Agricultural, forestry, hunting and fishing workers **(Skilled Agricultural, Forestry and Fishery Workers)**
2.	Construction worker	627	5.8	Workers in the production of industrial goods and services **(Craft and Related Trades Workers)**
3.	Recyclable material collector	493	4.6	Service workers, salespeople in stores and markets**(Craft and Related Trades Workers)**
4.	Multi-purpose agricultural producer	295	2.7	Agricultural, forestry, hunting and fishing workers **(Skilled Agricultural, Forestry and Fishery Workers)**
5.	Flying agricultural worker	285	2.6	Agricultural, forestry, hunting and fishing workers **(Skilled Agricultural, Forestry and Fishery Workers)**

6. Student	216	2.0	Outside the labour force (Non-economically active population)
7. Rice cultivation worker	198	1.8	Agricultural, forestry, hunting and fishing workers **(Skilled Agricultural, Forestry and Fishery Workers)**
8. Construction contractor	155	1.4	Workers in the production of industrial goods and services **(Craft and Related Trades Workers)**
9. Housekeeper (agricultural)	144	1.3	Agricultural, forestry, hunting and fishing workers **(Skilled Agricultural, Forestry and Fishery Workers)**
10. Retired person	141	1.3	Outside the labour force (Non-economically active population)
Others	3,851	64.2	
Total	10,769	100	

Supplementary Table 4. Resident population and employed labor force per Brazilian region, according to the Brazilian Institute of Geography and Statistics (2022).

	Resident Population	Labor force
Regions		
Northern	17,354,884	8,157,000
Northeastern	54,658,515	22,481,000
Southeastern	84,840,113	44,429,000
Southern	29,937,706	15,813,000
Central-Western	16,289,538	8,490,000

Total of Brazil	203,080,756	99,370,000

Supplementary Table 5. Persons in labor age 14 or over, employed in the reference week, by occupational group in the main job, Brazil and Greater Region.

Main Groups	Brazil	Northern	Northeastern	Southeastern	Southern	Central-Western
1- Senior members of the public authorities, directors of public interest organizations and companies, managers **(Managers)**	3,349,000	192,000	437,000	1,751,000	653,000	317,000
2- Professionals in the sciences and arts **(Professionals)**	12,351,000	771,000	2,285,000	6,470,000	1,854,000	970,000
3- Middle-level technicians**(Technicians and Associate Professionals)**	8,247,000	511,000	1,500,000	4,165,000	1,405,000	666,000
4- Administrative service workers **(Technicians and Associate Professionals)**	8,309,000	571,000	1,636,000	3,949,000	1,324,000	828,000
5- Service workers, salespeople in stores and markets**(Craft and Related Trades Workers)**	22,339,000	1,965,000	5,735,000	9,808,000	2,907,000	1,925,000
6- Agricultural, forestry, hunting and fishing workers **(Skilled Agricultural, Forestry and Fishery Workers)**	5,381,000	841,000	1,661,000	1,334,000	1,192,000	353,000
7- Workers in the production of industrial goods and services **(Craft and Related Trades Workers)**	13,298,000	1,003,000	2,718,000	5,971,000	2,452,000	1,154,000
8- Workers in the production of industrial goods and services **(Plant and Machine Operators and Assemblers)**	9124000	714,000	1,857,000	4,107,000	1,670,000	776,000
9-Repair and maintenance workers **(Elementary Occupations)**	16,129,000	1,502,000	4,474,000	6,539,000	2,235,000	1,379,000
0-Members of the lawful forces armed forces, police and military firemen **(Armed Forces Occupations)**	832,000	87,000	176,000	327,000	121,000	121,000
Total	99,370,000	8,157,000	22,481,000	44,429,000	15,813,000	8,490,000

Supplementary Table 6. Results of Linear Mixed Model (LMM) comparing the occurrence of leptospirosis in the labor population of the 100 biggest Brazilian cities, in function of the spatial distribution (each geographical region) and the susceptibility to disease promoted by work groups.

**Explanatory variables**	**Test**	**Estimate**	**S.E.**	**t-value**	**p-value**
Brazilian regions	Midwest X North	-4.70E-06	1.92E-06	-2.446	0.1122
	Midwest X Northeast	-2.37E-06	1.67E-06	-1.413	0.6208
	Midwest X South	-7.18E-06	1.76E-06	-4.069	0.0009*
	Midwest X Southeast	-1.13E-06	1.54E-06	-0.736	0.9476
	North X Northeast	2.33E-06	1.53E-06	1.525	0.5491
	North X South	-2.48E-06	1.63E-06	-1.523	0.5501
	North X Southeast	3.57E-06	1.38E-06	2.584	0.0814
	Northeast X South	-4.81E-06	1.33E-06	-3.624	0.0042*
	Northeast X Southeast	1.23E-06	1.01E-06	1.224	0.7377
	South X Southeast	6.05E-06	1.15E-06	5.247	<.0001*
Occupational Susceptibility	Average X High	-7.92E-06	5.86E-07	-13.521	<.0001*
	Average X Low	-2.20E-08	5.86E-07	-0.038	0.9992
	High X Low	7.90E-06	6.26E-07	12.613	<.0001*

* Statistical significance.

Supplementary Table 7A. Distribution of severe clinical manifestations, hospitalization rates, and case fatality rates by (BIGS/COC) occupational groups in confirmed leptospirosis cases (≥14 years), Brazil, 2007–2022.

Occupational Group (BIGS/COC)	Frequent Occupations (examples)	Renal Failure (%)	Jaundice (%)	Pulmonary Hemorrhage (%)	Hospitalization (%)	Case Fatality (%)
1 – Public administration / managers	Public managers, directors	61.1	21.0	43.1	71.1	8.6
2 – Science and arts professionals	Physicians, teachers, engineers	49.6	16.1	35.3	57.2	4.4
3 – Mid-level technicians	Administrative technicians, lab assistants	57.4	20.0	47.4	70.7	7.1
4 – Administrative service workers	Clerks, office assistants	58.6	19.7	41.0	64.2	5.8
5 – Service workers / commerce	Recyclable material collectors, salespeople	65.0	21.3	56.6	74.7	10.7
6 – Agricultural / forestry / fishing	Rural workers, caretakers	60.3	19.4	41.4	69.2	4.7
7 – Industrial production workers	Bricklayers, construction assistants	60.6	21.0	55.6	76.9	10.5
8 – Plant and machine operators	Production line operators	59.4	24.6	48.7	73.2	7.7
9 – Maintenance / repair workers	Mechanics, electricians	59.4	24.6	48.7	73.2	7.7
0 – Armed forces / police / firefighters	Soldier, military police, firefighter	36.2	19.5	25.2	62.9	3.3

Supplementary Table 7B. Ranking of specific occupations (CBO) with the highest risk of lethality and hospitalization in confirmed leptospirosis cases (≥14 years), Brazil, 2007–2022.

Rank	CBO Code	Occupation	Cases	Hospitalization (%)	Case Fatality (%)
1	519205	Recyclable material collector	712	80.8	14.9
2	715210	Bricklayer	2,116	80.2	12.9
3	717020	Construction assistant	490	80.8	9.4
4	622005	Agricultural caretaker	332	80.1	9.9
5	512105	Domestic worker	268	71.3	11.2
6	782510	Truck driver (regional/international routes)	367	74.7	6.5
7	622020	Temporary agricultural worker	596	72.8	5.7
8	141410	Retail trader	246	71.5	8.5
9	621005	General agricultural worker	2,255	69.0	3.9

Rank	CBO Code	Occupation	Cases	Hospitalization (%)	Case Fatality (%)
10	612005	Multipurpose agricultural producer	551	67.2	3.8
